# End-to-side neurorrhaphy in the reconstruction of peripheral segmental neural loss: an experimental study

**DOI:** 10.1590/acb394024

**Published:** 2024-07-22

**Authors:** Rafael Silva Lemos, Livia Guerreiro de Barros Bentes, Maria Eduarda dos Santos Lopes Vasconcelos, Daniela Ferreira Tramontin, Luís Vinícius Pires da Costa, Antonio Leonardo Jatahi Cavalcanti Pimentel, Nayara Pontes de Araújo, Mariseth Carvalho de Andrade, Danusa Neves Somensi, Rui Sérgio Monteiro de Barros

**Affiliations:** 1 Universidade do Estado do Pará – Faculdade de Medicina – Laboratório de Cirurgia Experimental – Belém (PA) – Brazil.; 2 Centro Universitário do Pará – Faculdade de Medicina – Laboratório de Cirurgia Experimental – Belém (PA) – Brazil.; 3 Universidade Federal do Pará – Faculdade de Medicina – Laboratório de Cirurgia Experimental – Belém (PA) – Brazil.; 4 Centro Universitário da Amazônia – Laboratório de Cirurgia Experimental – Belém (PA) – Brazil.

**Keywords:** Median Nerve, Microsurgery, Nerve Regeneration, Rats, Wistar

## Abstract

**Purpose:**

To evaluate the effects on peripheral neural regeneration of the end-to-side embracing repair technique compared to the autograft repair technique in Wistar rats.

**Methods:**

Fifteen male Wistar rats were divided into three groups with five animals each: denervated group (GD), autograft group (GA), and embracing group (EG). For the evaluation, the grasping test, electroneuromyography (ENMG), and muscle weight assessment were used.

**Results:**

Muscle weight assessment and ENMG did not show significant neural regeneration at the end of 12 weeks in the DG and GE groups, but only in GA. The grasping test showed an increase in strength between the surgery and the fourth week in all groups, and only the GA maintained this trend until the 12th week.

**Conclusions:**

The present study indicates that the neural regeneration observed in the end-to-side embracing neurorrhaphy technique, in the repair of segmental neural loss, is inferior to autograft repair in Wistar rats.

## Introduction

Peripheral nerve injuries can occur in diverse ways, the main ones being crushing, or compression and transection caused by sharp objects^1^. Most injuries occur due to accidents, mostly affecting men of working age from 10 to 69 years old, in work, automobile and sports environments^1^^,^ 2, causing major functional and socioeconomic repercussions^2^^,^ 3.

Unlike the central nervous system, the peripheral nerve can regenerate after injury. There are several factors, both intrinsic–including the patient’s age, type and diameter of the nerve, and the ability of Schwann cells to multiply–and extrinsic, such as the extent of the injury, surgical repair technique, and post-operative care as physiotherapy and rehabilitation4. These promote axon regeneration through sites of injury and then reestablish functional connections with their original target organs^4^.

Ten percent of hand injuries are accompanied by neural injuries, and the digital nerves are most often injured. Among these, 18% have segmental neural loss and require nerve grafts^5^. Primary end-to-end neurorrhaphy is the preferred repair method when appropriate^6^.

Strategies to reconstruct a segmental neural loss may include primary repair performed under excessive tension with restriction of joint movement; the use of hollow conduit to fill the gap; autologous nerve graft; allograft, end-to-end repair and neural transfer^7^.

The adverse influence of tension on nerve regeneration was initially observed by Duarte-Moreira et al.^6^, and excessive tension at the repair site is directly proportional to the proliferation of fibrous tissue at the repair site, negatively influencing neural regeneration^4^. The use of conduits for segmental losses results in inferior results, but the use of conduits as an internal splint to relieve in-situ resting stress is promising^7^.

Autologous nerve grafting has historically been the most reliable method, the gold standard, but this alternative requires an additional incision to extract the graft, which can cause significant morbidity in the donor area, such as areas of insensitivity and the formation of neuromas^5^^,^ 8. Advances in tissue engineering in the last decade have allowed the production of decellularized nerve allografts that, unlike conventional allografts, are depurated of their antigenic component, but which still maintain their three-dimensional structure of the nerve skeleton for axonal growth. Decellularized nerve allografts represent a promising alternative with the potential to be explored^9^, but they have an exorbitant cost^10^^–^ 13.

In the last 20 years, the alternatives for reconstructing peripheral neural injuries that cannot be approximated by coaptation without tension have expanded. In proximal injuries, nerve transfers are increasingly being performed. However, in distal injuries, interposition nerve grafting remains the most used method^5^. Therefore, with the limitations of current techniques, there is an urgent requirement to develop nerve repair techniques.

Thus, the aim of this study was to evaluate the embracing type of end-to-side repair method in the treatment of peripheral segmental neural loss in comparison with the autologous graft in Wistar rats.

## Methods

This study was approved by the Ethics Committee on the Use of Animals of the Universidade do Estado do Pará (UEPA), under protocol no. 50/20, and followed the rules of Brazilian national legislation for the use of animals (Law no. 11,794/08), which is based on the National Institutes of Health protocol, and the ethical code of the Council for the International Organization of Medical Sciences for animal experimentation and the Animal Research: Reporting of In Vivo Experiments (ARRIVE) guidelines.

Fifteen male Wistar rats (*Rattus norvegicus*) were used, 12 weeks old and weighing between 150–200 grams, without veterinary diseases. The animals were kept in the vivarium of the UEPA Experimental Surgery Laboratory, in Brazil, in an environment with controlled light, temperature, humidity, and noise, with water and food *ad libitum*. Non-sterile polyurethane cages with capacity for three or four rats each were used, lined with sterile wood shavings. No type of environmental enrichment was added.

The animals were randomly divided into three groups with five animals each: denervated group (GD), autograft group (GA), and embracing group (GE).

Initially, anesthesia was performed in all animals through intraperitoneal injection of 70 mg/kg of ketamine hydrochloride and 10 mg/kg of xylazine hydrochloride. To confirm the anesthetic plan, the loss of caudal, foot, and vibrissae reflexes were verified. Subsequently, epilation and antisepsis of the right anterior limb were performed, and the animals were positioned in dorsal decubitus with the limbs fixed in abduction.

### Surgical procedure

A 3-cm longitudinal incision was made on the medial aspect of the brachial segment of the right forelimb. The anterior brachial muscles and triceps were moved apart to allow visualization of the median and ulnar nerves separated by the brachial arteriovenous bundle.

**Figure 1:**
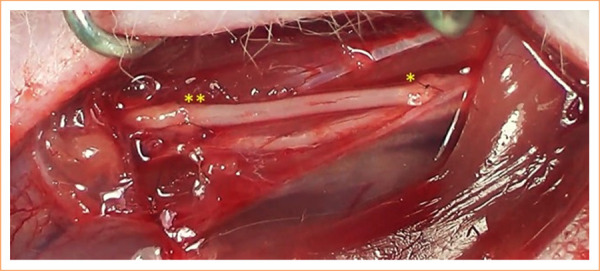
Median nerve repair using the autograft technique. In this technique, the nerve segment was sectioned, and its direction was reversed.

**Figure 2:**
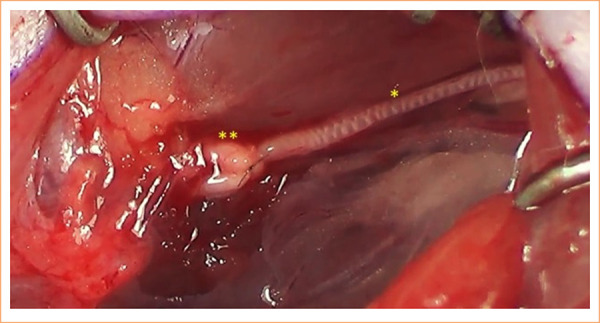
Repair of the median nerve with the ulnar nerve using the embracing technique.

**Figure 3:**
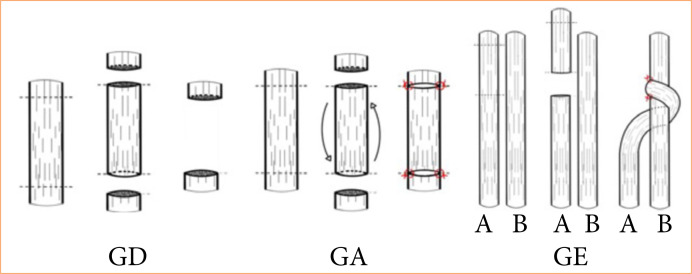
Demonstration of the techniques performed.

DG: denervated group, with the removal of 10 mm of length of the median nerve, without performing neurorrhaphy; GA: the excised segment was inverted and used as an autograft and repaired with 10-0 nylon monofilament suture using two simple sutures, both proximally and distally; GE: (A) median nerve had its proximal segment removed and discarded, with the distal stump embracing (B) the ulnar nerve and being sutured anteriorly to the epineurium of the ulnar nerve. Source: Elaborated by the authors.

In all groups, an excision of a 10-mm segment of the median nerve was performed. In the GA group, the excised segment was inverted and used as an autograft and repaired with 10-0 nylon monofilament suture using two simple sutures, both proximally and distally (Fig. 1). In the GD group, the removed segment was discarded, leaving a gap of 10 mm in the median nerve. In the GE group, the distal stump of the median was brought behind the ulnar nerve, from lateral to medial, and then brought around the ulnar from back to front, from medial to lateral, thus embracing the ulnar nerve and, finally, sutured anteriorly to the epineurium of the ulnar nerve without sectioning it, with 10-0 nylon monofilament thread (Fig. 2). The three surgical techniques can be seen in Fig. 3.

*proximal stump; **distal stump. Source: Elaborated by the authors.

*ulnar nerve; **the medial nerve repaired through the embracing technique surrounding the median nerve. Source: Elaborated by the authors.

At the end of the procedures, the skin and subcutaneous tissue were sutured with 5-0 nylon monofilament.

All surgical procedures were performed in a single day by a surgeon with more than 20 years of experience in microsurgery and accompanied by a veterinarian.

**Figure 4:**
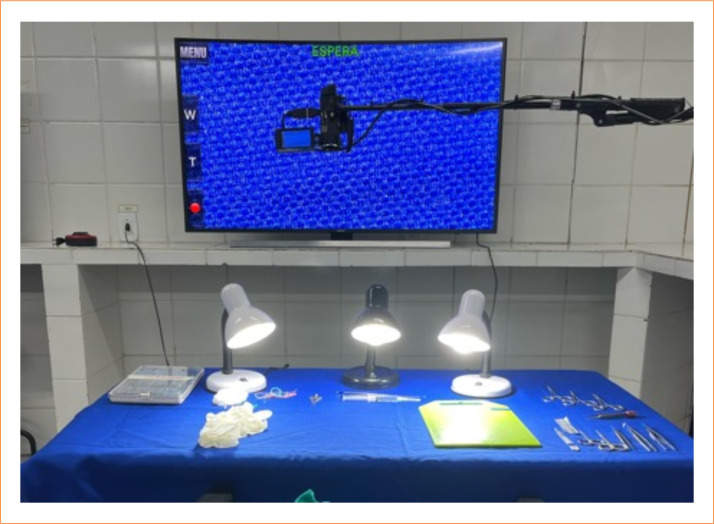
Videomagnification system.

Source: Archive of the Microsurgery Line of the Experimental Surgery Laboratory of Universidade do Estado do Pará.

To better visualize the anatomy during the operations, the video magnification system developed by the research group was used (Fig. 4), consisting of a Sony© Handycam HDR-XR160 camera connected to a 55’ Curve Full HD TV using an HDMI cable, providing a magnification of image of 56 times^14^^,^ 15. Three white halogen lamps were used to illuminate the surgical field.

### Group analysis

#### Grasping test

For the functional analysis of the animal’s digital grip strength, the grasping test^16^ was used, in which the rat is held by the examiner supported on its hind legs while the forelimb is positioned on a pressure dynamometer. Next, the grip stimulus is stimulated by slight traction on the animal’s tail, and the maximum force exerted by the rat is recorded.

**Figure 5:**
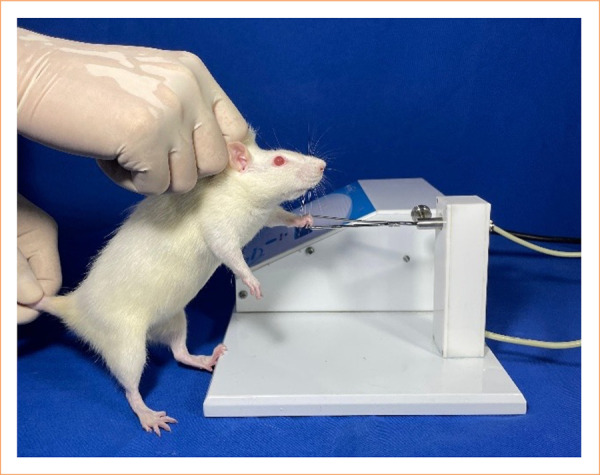
Grasping test being performed.

Source: Archive of the Microsurgery Line of the Experimental Surgery Laboratory of Universidade do Estado do Pará.

This test was performed three times on both forelimbs of the animals: preoperatively, four and 12 weeks postoperatively. To this end, the BS-GRIP Grip Meter^®^ dynamometer was used, and the force measurement was recorded in grams. For measurement, each rat limb was evaluated three times, with the measurement having the highest value for analysis being considered (Fig. 5).

### Electroneuromyography

For the electrophysiological evaluation of the animals, electroneuromyography of the animals’ right anterior limb was also used at three moments: preoperatively, four and 12 weeks postoperatively.

During the procedure, the room was maintained at 25ºC, and anesthesia was performed with an intraperitoneal injection of ketamine hydrochloride and xylazine hydrochloride. Afterwards, epilation and antisepsis were performed on the right anterior limb and abdomen of the animals.

The animals were positioned in dorsal recumbency with the right forelimb fixed. Then, the ground electrode was positioned on the abdomen, and the needle and reference electrodes were connected to the flexor carpi radialis muscle and its tendon, respectively. The pulses were generated through a bipolar electrode, in which the anode and cathode were 1 mm apart. The stimulus used varied depending on the response of each animal.

For analysis, the latency and amplitude of median nerve action potentials were recorded. The Medelec/TECA–Synergy EMG System N2 electroneuromyography (ENMG) was used.

### Weight and muscles assessment

The weight of the animals was measured before surgery and 12 weeks postoperatively, using a precision digital scale. In the 12th week, after analyzing the grasping test and ENMG, the rats were euthanized with triple the anesthetic dose of ketamine hydrochloride and xylazine hydrochloride intraperitoneally. Next, the pronator teres, flexor carpi ulnaris, flexor carpi radialis, palmaris longus, and flexor digitorum superficialis muscles were collected and weighed on a precision scale.

### Statistical analysis

The data obtained were organized and tabulated with the Microsoft Excel 2020 and Microsoft Word 2020 softwares. For statistical analysis, the analysis of variance (ANOVA) and T-student’s tests were performed with the Biostat 5.3® program, adopting the 95%-confidence interval and the significant value of p < 0.05.

## Results

During the study period, no animal died or showed signs of illness or postoperative complications during the research period.

Regarding the body weight of the animals, there was no statistically significant difference between the groups over the weeks. The average preoperative weight, at four and 12 weeks was: 337.2; 396; and 490.4 grams in the GD group, 367.6; 511.2; and 522.4 grams in the GA group, and 490.2; 502.2; and 530.2 grams in the GE group (p > 0.05 in the ANOVA tests and paired t-test between groups).

### Grasping test

Table 1 describes the means and standard deviations of the GD, GA, and GE groups in the grasping test.

**Table 1:** Grasping test of the left and right forelimbs of the animals during the studied period!.

Grasping Left Forelimb	Denervated Group				Autograft Group				Embracing Group		
	Week 0	Week 4	Week 12		Week 0	Week 4	Week 12		Week 0	Week 4	Week 12
Minimum	206	120	182		232	127	230		234	131	152
Maximum	400	422	403		344	285	420		344	459	523
Average	294.4	222.2	265.2		265.0	345.8	315.2		274.0	265.2	314.0
Standard deviation	75.36	120.43	89.92		47.13	140.79	88.25		43.40	135.01	156.47
Between weeks*	*p* = 0.5194				*p* = 0.5399				*p* = 0.8015		
Minimum	197	7	0		248	0	194		248	0	0
Maximum	345	15	96		424	79	336		424	121	37
Average	269.8	12.0	37.0		340.8	56.8	237.6		363	55.0	15.2
Standard deviation	69.65	3.16	50.72		81.38	32.84	61.41		73.72	43.86	16.08
Between weeks	*p* < 0.0001*				*p* < 0.0001*				*p* < 0.0001*		
Week 0 × Week 4	*p* < 0.0010**				*p* < 0.0010**				*p* < 0.0010**		
Week 0 ×Week 12	*p* < 0.0010**				*p* = 0.0216**				*p* < 0.0010**		
Week 4 × Week 12	*p* > 0.05				*p* < 0.0010**				*p* > 0.05		

* Analysis of variance one criterion;

** paired t-test;

! all values in gram-force.

Source: Elaborated by the authors.

### Electroneuromyography

Table 2 describes the findings relating to ENMG of the ulnar nerve in the GD, GA, and GE groups, while Table 3 correlates them to the median nerve.

**Table 2:** Amplitude and latency of ulnar nerve electroneuromyography during the studied period!.

Ulnar Amplitude	Denervated Group			Autograft Group			Embracing Group	
	Week 4	Week 12		Week 4	Week 12		Week 4	Week 12
Minimum	2.0	4.8		8.6	7.7		1.0	1.7
Maximum	5.5	9.6		30.1	35.4		1,4	6.6
Average	3.8	7.0		22.8	26.7		1.2	3.4
Standard deviation	1.360	2.056		8.868	11.854		0.215	2.115
Between weeks	*p* = 0.0331*			*p* = 0.1850*			*p* = 0.0316*	
**Ulnar latency**	**Denervated Group**			**Autograft Group**			**Embracing Group**	
	**Week 4**	**Week 12**		**Week 4**	**Week 12**		**Week 4**	**Week 12**
Minimum	1.1	1.2		0.8	1.0		1.0	1.1
Maximum	1.5	1.7		1.4	2.1		1.4	2.3
Average	1.2	1.5		1.0	1.8		1.2	1.5
Standard deviation	0.187	0.185		0.243	0.476		0.215	0.470
Between weeks	*p* = 0.0122*			*p* = 0.0063*			*p* = 0.1739*	

* Paired t-test;

! amplitude expressed in mV and latency in ms.

Source: Elaborated by the authors.

**Table 3:** Amplitude and latency of median nerve electroneuromyography during the studied period!.

Median amplitude	Denervated Group			Autograft Group			Embracing Group	
	Week 4	Week 12		Week 4	Week 12		Week 4	Week 12
Minimum	7.4	2.1		7.1	8.7		1.1	1.0
Maximum	13.0	13.5		21.2	26.8		1.2	13.3
Average	9.4	7.0		16.5	20.0		1.1	5.4
Standard deviation	2.200	4.678		5.649	7.311		0.073	5.232
Between weeks	*p* = 0.1417*			*p* = 0.1096*			*p* = 0.0705*	
**Median latency**	**Denervated Group**			**Autograft Group**			**Embracing Group**	
	**Week 4**	**Week 12**		**Week 4**	**Week 12**		**Week 4**	**Week 12**
Minimum	1.0	1.2		1.3	1.2		1.1	1.3
Maximum	1.5	2.1		1.4	1.8		1.2	1.6
Average	1.2	1.6		1.3	1.4		1.1	1.4
Standard deviation	0.184	0.386		0.042	0.250		0.073	0.120
Between weeks	*p* = 0.0262*			*p* = 0.2581*			*p* = 0.0219*	

* Paired t-test;

! amplitude expressed in mV and latency in ms.

Source: Elaborated by the authors.

### Muscle weight assessment

Tables 4 and 5 describe the findings regarding the weight of the animals’ muscles at the end of the study.

**Table 4:** Weight of the flexor carpi ulnaris, palmar longus, and flexor carpi radial muscles!.

Muscle weight	Ulnar carpal flexor				Long palm				Radial carpal flexor		
	Denervated	Autograft	Embracing		Denervated	Autograft	Embracing		Denervated	Autograft	Embracing
Minimum	0.151	0.163	0.16		0.025	0.048	0.024		0.025	0.058	0.022
Maximum	0.232	0.197	0.24		0.064	0.068	0.046		0.049	0.089	0.051
Average	0.1972	0.1808	0.205		0.0378	0.0578	0.0332		0.0338	0.0722	0.031
Standard deviation	0.0298	0.0154	0.0306		0.0165	0.0074	0.008		0.0091	0.0124	0.0126
Between weeks	*p* = 0.3622*				*p* = 0.0120*				*p* = 0.0003*		
Denervated × Autograft	NA				*p* = 0.0171**				*p* < 0.0010**		
Denervated × Embracing	NA				*p* > 0.05				*p* > 0.05		
Autograft × Embracing	NA				*p* = 0.0053**				*p* < 0.0010**		

* Paired t-test;

! amplitude expressed in mV and latency in ms.

Source: Elaborated by the authors.

**Table 5:** Weight of the superficial muscles of the fingers and pronator teres!.

Muscle weight	Superficial finger				Round prong		
	Denervated	Autograft	Embracing		Denervated	Autograft	Embracing
Minimum	0.083	0.204	0.093		0.071	0.071	0.083
Maximum	0.129	0.245	0.154		0.099	0.094	0.106
Average	0.1034	0.2242	0.1112		0.0862	0.0854	0.0984
Standard deviation	0.0221	0.0192	0.025		0.0121	0.0091	0.0099
Between weeks	*p* < 0.0001*				*p* = 0.0128*		
Denervated × Autograft	*p* < 0.0010**				NA		
Denervated × Embracing	*p* > 0.05				NA		
Autograft × Embracing	*p* < 0.0010**				NA		

* Analysis of variance one criterion;

** paired t-test;

NA: not applicable;

! all values in grams.

Source: Elaborated by the authors.

## Discussion

Injuries to peripheral nerves can occur in diverse ways, the main ones being traumatic in origin due to sharp objects and stretching, with consequent disruption of the nervous tissue. Based on the type of injury and the size of the injured area, different surgical treatment modalities can be addressed. The techniques of microsurgical suture are vast, covering end-to-end, end-to-side neurorrhaphies–this, using various techniques that will be discussed later–, and the use of conduits made of varied materials (organic and inorganic). According to current literature, the superiority of the autograft technique is undeniable, and it is currently considered the gold standard. However, this technique has limitations in correcting extensive lesions^1^^–^ 3 ^,^ 17 ^,^ 18. In this case, the development of techniques, such as embracing end-to-side neurorrhaphy, is significant to overcome such problems (Fig. 3).

Regarding the weight of the rats, there was an increase in the total average over the weeks due to the natural growth of the animals. In this sense, as there was no statistical difference in the comparison between the groups, the homogeneity of the study sample is reliable.

Concerning the grasping test, about the operated limb, the GA group showed a significant improvement and recovery of the strength of the operated limb, which was already expected as it is the actual gold standard treatment in these situations^13^. In the GE and GD groups, lower grip strength was observed in the grasping test, as well as a lack of statistical improvement between the 4th and 12th week. The strength of the non-operated left forelimb was similar in all groups, with no significant differences over the study weeks, as shown in Table 2.

When comparing DG and GE, it appears that, in the 4th post-operative week, the GD had lower strength (average of 12 g) and the GE had greater recovery (average of 55 g). However, in the 12th week, after eight weeks, the GD had an increase in strength, while the GE showed a decrease and absence of strength, but the variation between the weeks did not demonstrate significance, due to the large variation in strength between the groups in the same week. This finding may have occurred due to the progression of Wallerian degeneration, which occurs through the progressive ascending denervation of the injured neural stump, but such findings could only be confirmed through histopathological analysis^11^^,^ 12.

ENMG was used to compare nerve conduction between groups, over four and 12 weeks, analyzing the degree of reinnervation over time. The amplitude assessment indicates the number of functioning nerve fibers and motor units recruited to conduct the electrical stimulus through the axonal fibers^18^^–^ 21. In the present study, there was a significant increase in the amplitude of the ulnar nerve in the 4th and 12th weeks, being greater in GA, with significant elevations in GE and GD, the latter being greater. Therefore, GA showed a greater recruitment and activation of nerve fibers, when compared to the other groups, as shown in Table 3. About the GE, no impairment in the function of the ulnar nerve was identified.

Relating the amplitude of the median nerve, a significant increase was observed over the weeks in GA and GE, with the latter having the lowest total values, while in the GD there was a small decrease in amplitude from the 4th to the 12th week, but still higher than the GE. This demonstrates an increase in the activation of motor units in all experimental groups^21^, despite GD having decreased, in Table 4. This corroborates the findings of the grasping test.

Regarding latency, this parameter allows checking the time needed for the action potential to travel a certain length of fibers, a direct product of myelination and the diameter of axonal fibers, wherefore the shorter the latency, the better the quality of neural regeneration^18^^-^ 21. When dealing with GD and GE, both presented an increase in latency between the 4th and 12th week, and in the GE the increase was minimally greater than in the GD, in both the median and ulnar nerves. Meanwhile, in GA the latency in the 4th week was higher than in other groups in the same periods, with higher values in the 12th week. This observation suggests that the ENMG had difficulty evaluating neural regeneration in terms of latency^19^^,^ 22.

Both the ENMG and the weight assessment of the muscle did not show, at the end of the 12th week, favorable statistical differences for neural regeneration, about the data obtained in the 4th week. Such observations suggest that the functional grasping test has greater sensitivity than the ENMG and the weight assessment of the muscle in quantifying signs of neural regeneration after neurorrhaphy^21^^,^ 22.

Among the muscles analyzed, the flexor carpi ulnaris, flexor carpi radialis, digitorum superficialis, and palmaris longus were the most important for the grip strength of the limb and the ones which had the greatest difference between the groups^21^^,^ 23 ^,^ 24. According to the weight of each muscle, it was possible to verify the difference in atrophy between the groups, demonstrating less loss of muscle mass in GA when compared to the other groups, which corroborates the functional findings of the grasping test (Tables 4 and 5).

Both GD and GE had lower weights, but the GE had a minimally greater mass, but without a statistically relevant difference, which indicates that they maintained trophism. In the study by Viterbo et al.11, different methods of end-to-side neurorrhaphy were evaluated, but they had equivalent results, better than the denervated groups, unlike our results, which were inferior. In their study, Viterbo et al.^11^ performed the embracing technique with a longitudinal incision in the free stump, so that the two epineural ends formed two borders that, when involving the donor’s nerve, were sutured together, whereas, in the present study, complete involvement of the free stump was performed to the donor’s nerve.

In the study by Zhao et al.^25^, it was proven, histologically, that end-to-side neurorrhaphy techniques with or without epineurectomy provide axonal growth, in diverse ways, the first with the diffuse growth of nerve fibers penetrating the epineurium and the second with growth directed through the created epineural window. In the present study, it was decided to perform embracing neurorrhaphy without epineurectomy because it is an innovative technique, however, if epineurectomy had been performed, GE could have obtained better functional results, by providing greater density of nerve fibers.

It should be noticed that the principle of the embracing technique is that it would provide a greater contact surface between the nerves, and it was expected that this would favor the action of Schwann cells and neural regeneration (Fig. 3).

Regarding this, the end-to-side and end-to-end neurorrhaphy techniques (this one used in GA) have a smaller area of contact with the donor nerve or nerve segments than the embracing technique used in the study, as its area refers only to the internal surface of the sectioned nerve, while the area of the embracing technique corresponds to the contour of the nerve around the perimeter of the donor nerve, thus having a greater area of intimate contact with the donor nerve.

However, as there were no significant benefits from this technique compared to the denervated group, we assumed that embracing neurorrhaphy may have, due to the compression of one nerve over the other, reduced circulation and nutrition to the nerves and, thus, had a negative effect in regeneration. Furthermore, the presence of the epineurium may have hindered the penetration of nerve fibers. Therefore, in this study, it was verified that the embracing technique is not superior to the current gold standard, which is the autograft.

It is worth highlighting that, although this study was experimental and, therefore, controlled, it has limitations. One of the main ones is the sample size, with five specimens per group, which can reduce the statistical accuracy of the data found. Furthermore, there was no assessment of the sensitivity of the animals due to the lack of techniques that provide this study, as well as histological analysis of the nervous tissue. With the above, it is proposed that such variables be considered for future studies.

## Conclusion

The results of the present study suggest that the embracing end-to-side repair method for the treatment of peripheral segmental neural loss in Wistar rats is inferior to autograft repair.

## Data Availability

All dataset were generated or analyzed in the current study.
